# Socioeconomic inequalities in HIV/AIDS mortality in urban areas of three Spanish cities

**DOI:** 10.1590/0102-311XEN141925

**Published:** 2026-03-23

**Authors:** Pamela Pereyra-Zamora, Andreu Nolasco, Javier Casillas-Clot, Nayara Tamayo-Fonseca

**Affiliations:** 1 Departamento de Enfermería Comunitaria, Medicina Preventiva y Salud Pública e Historia de la Ciencia, Universidad de Alicante, San Vicente del Raspeig, España.

**Keywords:** Social Deprivation, Social Inequalities, Mortality, HIV, AIDS, Privación Social, Desigualdades Sociales, Mortalidad, VIH, SIDA, Privação Social, Desigualdades Sociais, Mortalidade, HIV, AIDS

## Abstract

This study analyzed socioeconomic inequalities in three Spanish cities (Alicante, Castellón, and Valencia), according to the level of deprivation of small urban areas, and assessed their impact on mortality due to HIV and AIDS. This ecological study used census tracts as the unit of analysis. A deprivation index score, based on employment, education, and housing indicators, was calculated for each census tract. Mortality rates were calculated by sex, age group, level of deprivation, and period (2000-2015), and relative risks were estimated. Between 2000 and 2015, 967 deaths related to HIV and AIDS were recorded in the three cities, with a substantial reduction in mortality from 600 deaths in 2000-2007 to 340 in 2008-2015, especially among people aged 0 to 44 years. Mortality remained consistently higher among men and in areas with greater socioeconomic deprivation. The results show that, despite the overall decline in mortality, living in deprived urban areas continues to be a key determinant of deaths due to HIV and AIDS. The study provides new evidence on the persistent impact of structural social inequalities on avoidable mortality in urban settings, even in a context of general epidemiological improvement.

## Abstract (es)

Este estudio analizó las desigualdades socioeconómicas en tres ciudades españolas (Alicante, Castellón y Valencia) según el nivel de privación de las pequeñas áreas urbanas, y evaluó su impacto en la mortalidad por el VIH y el SIDA, así como su evolución tras la crisis económica de 2008. Se llevó a cabo un estudio ecológico utilizando los bloques censales como unidad de análisis. Se calculó una puntuación del índice de privación, basada en indicadores de empleo, educación y vivienda, para cada bloque censal. Se calcularon las tasas de mortalidad por sexo, grupo de edad, nivel de privación y período (2000-2015), y se estimaron los riesgos relativos. Entre 2000 y 2015, se registraron 967 muertes relacionadas con el VIH y el SIDA en las tres ciudades, con una reducción sustancial de la mortalidad, que pasó de 600 muertes en 2000-2007 a 340 en 2008-2015, especialmente entre las personas de 0 a 44 años. La mortalidad siguió siendo sistemáticamente más elevada entre los hombres y en las zonas con mayores carencias socioeconómicas. Los resultados muestran que, a pesar de la disminución general de la mortalidad, vivir en zonas urbanas desfavorecidas sigue siendo un factor determinante clave de las muertes por el VIH y el SIDA. El estudio aporta nuevas pruebas sobre el impacto persistente de las desigualdades sociales estructurales en la mortalidad evitable en contextos urbanos, incluso en un contexto de mejora epidemiológica general.

## Abstract (pt)

Este estudo analisou desigualdades socioeconômicas em três cidades espanholas (Alicante, Castellón e Valencia) de acordo com o nível de privação das pequenas áreas urbanas, e avaliou seu impacto na mortalidade devido ao HIV e à aids, bem como sua evolução após a crise econômica de 2008. Um estudo ecológico foi realizado utilizando setores censitários como a unidade de análise. O índice de privação, baseado em indicadores de emprego, educação e habitação, foi calculado para cada setor censitário. As taxas de mortalidade foram calculadas por sexo, faixa etária, nível de privação e período (2000-2015), com a estimativa de riscos relativos. Entre 2000 e 2015, foram registradas 967 mortes relacionadas ao HIV/aids nas três cidades, com redução substancial de 600 mortes em 2000-2007 para 340 em 2008-2015, especialmente entre pessoas de 0 a 44 anos. A mortalidade permaneceu consistentemente maior entre os homens e em áreas com maior privação socioeconômica. Os resultados mostram que, apesar da queda geral na mortalidade, viver em áreas urbanas carentes continua sendo um fator determinante das mortes devido ao HIV/aids. Este estudo contribui com novas evidências sobre o impacto persistente das desigualdades sociais estruturais na mortalidade evitável em contextos urbanos, mesmo em um contexto de melhora epidemiológica geral.

## Introduction

Since the onset of the AIDS pandemic, over 75 million people have been infected with the HIV, resulting in more than 32 million deaths due to HIV-related causes. As of 2024, an estimated 40.8 million people were living with HIV globally, with 9.4 million not receiving treatment. Despite HIV remaining a global health challenge, the number of HIV-related deaths has been consistently decreasing worldwide ^1^. However, the decline in mortality varies significantly depending on the region, country, or population ^2^.

The advent of highly active antiretroviral therapy (HAART) as a first-line treatment for HIV has been a significant factor in these trends. HAART not only improves the quality of life for infected people but has also extended post-infection life expectancy from 10-12 years to approximately 25 years ^3^ ^,^ ^4^.

The World Health Organization (WHO) has established the ambitious goal of ending AIDS as a public health threat by 2030 ^5^. Consistently, the Joint United Nations Programme on HIV/AIDS (UNAIDS) introduced the “95-95-95” targets for 2030, aiming to ensure that 95% of people living with HIV are aware of their status, 95% of those diagnosed are on treatment, and 95% of those treated achieve viral suppression ^6^ ^,^ ^7^. However, UNAIDS acknowledges that health care inequalities constitute a primary barrier to achieving these objectives ^1^. In fact, it is known that socioeconomic disparities play a critical role in the prevention, treatment, and outcomes of HIV infection ^8^ ^,^ ^9^. Furthermore, factors such as race, immigrant status, gender, and sexual orientation significantly influence prevention measures, treatment adherence, and health outcomes ^10^ ^,^ ^11^ ^,^ ^12^ ^,^ ^13^.

These inequalities have a particularly evident impact in urban settings ^14^ ^,^ ^15^. Several studies in high-income countries, including Spain, have shown that HIV distribution is largely determined by the direct environment in which people live ^9^ ^,^ ^16^ ^,^ ^17^. In Spain, within the framework of the MEDEAS-II project, a deprivation index was designed to measure the socioeconomic level of small geographical areas ^18^, which has proven very useful in the study of mortality inequalities ^19^ ^,^ ^20^ ^,^ ^21^. The study of mortality in small areas, using the deprivation index, in Alicante, Valencia, and Castellón, three cities on the Mediterranean coast in Spain, showed significant inequalities between different groups according to their socioeconomic status. Moreover, these inequalities persisted before and after the 2008 economic crisis ^22^.

In Spain, previous studies have examined socioeconomic inequalities in overall and cause-specific mortality using deprivation indices. However, to our knowledge, no studies have analyzed the long-term evolution of HIV/AIDS-specific mortality at the small-area level in association with deprivation indicators, which is crucial to better understand its social distribution. The analysis of HIV/AIDS mortality can serve as a vital indicator for evaluating the effectiveness of prevention plans and actions geared toward the disease. Furthermore, examining different settings and the socioeconomic and demographic characteristics of affected individuals can help identify the profiles of those most vulnerable to the disease.

This study aims to explore the influence of socioeconomic inequalities on HIV/AIDS mortality in three cities along the Spanish Mediterranean coast - Alicante, Valencia, and Castellón. The analysis focuses on differences across levels of socioeconomic deprivation in small urban areas, providing evidence on how structural inequalities influence HIV/AIDS mortality patterns.

## Methods

### Study design, population and analysis units

This ecological study analyzes mortality trends by comparing two distinct time periods: 2000-2007 and 2008-2015. The analysis units were the census tracts (CTs) of three cities in southeastern Spain, located along the Mediterranean coast in the Valencian Community: Alicante (178 CTs), Castellón (58 CTs), and Valencia (531 CTs).

During the first period (2000-2007), the average total population across these cities was 1,240,744 inhabitants, which increased to 1,310,123 inhabitants in the second period (2008-2015). Population data disaggregated by CT, year, age, and sex were obtained from the Valencian Institute of Statistics, the official body responsible for producing population statistics for the Valencian Community.

### Mortality data

All deaths that occurred among residents of Alicante, Castellón, and Valencia were included in the analysis. Mortality data were obtained from the Mortality Register of the Valencian Community and were disaggregated by year of death, age, sex, city of residence, and cause of death.

The study focused exclusively on deaths attributed to HIV/AIDS, identified using the International Classification of Diseases, 10th revision (ICD-10), codes B20 to B40 and R75. Deaths were considered across all age groups and were georeferenced to their corresponding CT of residence.

### Census tract socioeconomic deprivation level

For each period and city, a deprivation index (DI) was calculated for each CT using the following socioeconomic indicators: unemployment, manual work, temporary employment, insufficient education among young people (16 to 29 years old), and insufficient education in the general population. These indicators were derived from the *Population and Housing Censuses* conducted in 2001 (for the period of 2000-2007) and 2011 (for the period of 2008-2015). The use of these two census years as reference points follows common practice in studies on health care inequalities, as the relative socioeconomic position of CTs tends to remain stable over time; intercensal interpolation was not considered appropriate due to the lack of annual small-area data and the risk of generating unreliable estimates.

The adopted DI was developed within the framework of the MEDEA-III project, in which both the socioeconomic and mortality data of this study are included ^18^. For each period and city, the 10th (P10), 25th (P25), 75th (P75) and 90th (P90) percentiles for DI were estimated, classifying CTs into five deprivation levels (DL) according to their value: DL1 for DI values below P10 (lower deprivation); DL2 for DI values between P10 and P25; DL3 for DI values between P25 and P75; DL4 for DI values between P75 and P90; and DL5 for DI values above P90 (higher deprivation). This classification was designed to better quantify the risks associated with socioeconomic disparities, comparing the most advantaged areas (DL1) with the most deprived areas (DL5). Detailed information on the average values for the five socioeconomic indicators across the DLs, as well as the geographic distribution of deprivation levels within the cities, can be found in a previous publication ^22^.

### Data analysis

To examine the evolution of mortality risk over time, data were classified into two time periods: 2000-2007 (P1) and 2008-2015 (P2). Deaths were grouped into the following age intervals: 0-44, 45-64, and ≥ 65. First, we computed the proportion of deaths attributed to HIV/AIDS among all deaths within each sex, age group, deprivation level and time period stratum. Specific mortality rates were subsequently calculated by sex, age group, DL, and time period.

To estimate the relative risks (RRs) between the categories of the variables under study, Poisson regression models were applied, adjusting for the effects of city (using dummy indicators), age, DL, and period, and segregating by sex. Robust estimation was used to account for any potential overdispersion in the data. Poisson regression models were estimated with deprivation level as a categorical variable (reference: low deprivation, DL1-DL3) and with study period as a binary variable (reference: 2000-2007), stratified by sex and age group. This structure allowed for the estimation of RRs across deprivation categories and periods. Overdispersion was tested in the dependent variable and we found that the variance-to-mean ratio was close to 1, indicating that overdispersion was not severe. Therefore, Poisson regression with robust standard errors was considered the most appropriate specification.

Before estimating the RRs by DLs and by periods, an analysis was conducted to assess whether the effects of DL differed significantly by city. Multivariate models were adjusted to include the effects of city, DL, period, and age group. The interaction terms were not significant for either men (p = 0.35) or women (p = 0.51). Based on this result, RR estimates were calculated for the three cities collectively.

**Figure 1:**
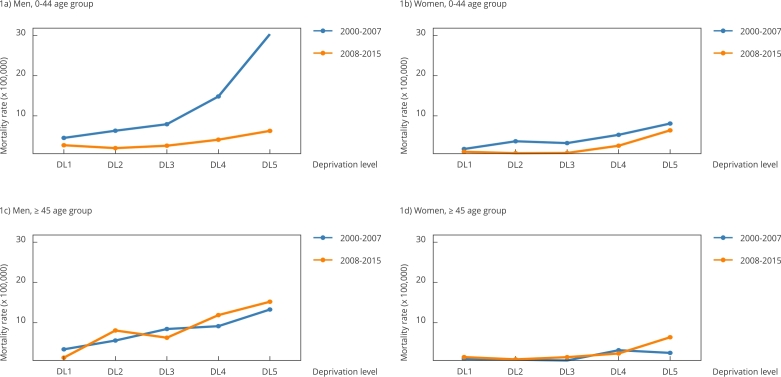
Specific mortality rates (per 100,000) due to HIV/AIDS, according sex, age group, deprivation level (DL), and period for all the cities studied. Cities of Alicante, Castellón and Valencia, Spain, 2000-2015.

Note: deprivation level (DL1 = least deprived, DL5 = most deprived).

In the combined analysis of the three cities, Poisson regression models indicated a second-level interaction between DL, period, and age group for both men and women. The graphical representations in Figure 1 suggest that this DL-Period-Age interaction may stem from different patterns of risk. In men, the interaction appears driven by the distinct risk patterns in the 0-44 age group, whereas, in women, it is associated with risk patterns in DL5 within the 0-44 and ≥ 65 age groups.

Given the presence of these interactions, RRs between DL categories (as a measure of inequalities) were estimated by sex, age, and period. Additionally, RR between periods (as a measure of temporal changes in the risk of death) were calculated, specific to age, sex, and DL.

For calculation of RR, the DL variable was grouped into two categories: low level of deprivation if DI ≤ P75 (including DL1, DL2 and DL3) and high level of deprivation if DI > P75 (DL4 and DL5). This categorization followed previous Spanish studies on mortality inequalities and was applied to ensure sufficient case numbers in each stratum. Age categories were regrouped into two intervals (0-44 and ≥ 45 years) due to the small number of HIV/AIDS deaths in some strata. The original age-disaggregated tables (with three categories) are provided in the Supplementary Material (https://cadernos.ensp.fiocruz.br/static//arquivo/suppl-e00141925_6071.pdf).

The statistical software SPSS v.25 (https://www.ibm.com/) was used for the computations, and figures were generated using R v.4.3.2 (http://www.r-project.org).

### Ethical approval

The data were obtained from secondary sources with anonymized databases; therefore, approval from an ethics committee is not required for this study.

## Results

Across the three cities, the reference population averaged 1,240,744 inhabitants per year in 2000-2007 and 1,310,123 in 2008-2015 (Supplementary Material - Table S1; https://cadernos.ensp.fiocruz.br/static//arquivo/suppl-e00141925_6071.pdf), with a stable sex distribution (48.2% men and 51.8% women in both periods). The largest age group was 0-44 years (59.9% in 2000-2007 and 56.6% in 2008-2015), followed by those aged 45-64 years (23.4% and 25.8%, respectively) and ≥ 65 years (16.7% and 17.6%). Regarding socioeconomic context, more than half of the population lived in areas with intermediate deprivation (DL3: 52.1% in 2000-2007; 52.5% in 2008-2015), while DL2 and DL4 represented moderate shares (16.5% and 14.5% in 2000-2007; 18.2% and 13.6% in 2008-2015). The extremes of deprivation accounted for smaller proportions (DL1: 8.0% and 7.0%; DL5: 8.9% and 8.7%, respectively).

Between 2000 and 2015, a total of 967 deaths due to HIV/AIDS were recorded. This corresponds to an average rate of 75.8 deaths per 100,000 inhabitants. Across the three cities: 206 in Alicante (64.6 per 100,000 inhabitants), 70 in Castellón (41.4 per 100,000 inhabitants), and 691 in Valencia (87.7 per 100,000 inhabitants). Among these, 27 deaths (2.8%) could not be georeferenced or assigned to a CT due to incomplete or incorrect address information, or because the reported residence fell outside the boundaries of the study cities.

Of the 940 georeferenced deaths, 600 occurred during the 2000-2007 period, while 340 were recorded between 2008 and 2015, reflecting a significant decrease in mortality over time. The average population in the studied cities was distributed across DLs, age groups, and sex, showing consistent patterns in both periods (2000-2007 and 2008-2015). The largest share of the population resided in areas with mid-level deprivation (DL3), with approximately 392,000 individuals aged 0-44 years (combining men and women) in both periods. The youngest age group (0-44 years) accounted for the majority of the population, followed by the 45-64 and ≥ 65 age groups. Slight variations were observed between sexes, with women generally outnumbering men in older age groups, particularly those aged ≥ 65, where women reached up to around 70,000 individuals in the second period. Overall, population sizes remained relatively stable across DLs and age groups between the two periods analyzed.

Table 1 summarizes the proportion of deaths attributable to HIV/AIDS by period, sex, and age group. A marked reduction in mortality was observed between the first and second periods, with a consistently higher frequency of deaths among men compared to women across all age groups and study periods. Notably, deaths due to HIV/AIDS were predominantly concentrated in the 0-44 age group for both sexes and study periods. Specifically, during the first period, 10.12% of male HIV/AIDS deaths and 8.09% of female HIV/AIDS deaths occurred in this age group, declining to 4.47% and 4.16%, respectively, in the second period.

**Table 1:** Frequencies and percentages of death due to HIV/AIDS deaths, according to age group, sex, and, period. Cities of Alicante, Castellón and Valencia, Spain, 2000-2015.

Period/Sex/Age group	HIV/AIDS deaths		Total deaths		% of deaths due to HIV/AIDS
	n	%	n	%	
2000-2007					
Male					
0-44 years	317	69.1	3,132	7.1	10.12
45-64 years	119	25.9	7,904	17.8	1.51
≥ 65 years	23	5.0	33,376	75.1	0.07
Total	459	100.0	44,412	100.0	1.03
Female					
0-44 years	114	80.9	1,410	3.4	8.09
45-64 years	25	17.7	3,500	8.3	0.71
≥ 65 years	2	1.4	37,157	88.3	0.01
Total	141	100.0	42,067	100.0	0.34
2008-2015					
Male					
0-44 years	92	36.9	1,940	4.4	4.74
45-64 years	139	55.9	7,688	17.5	1.81
≥ 65 years	18	7.2	34,214	78.1	0.05
Total	249	100.0	43,842	100.0	0.57
Female					
0-44 years	44	48.4	1,054	2.4	4.18
45-64 years	44	48.4	3,780	8.5	1.16
≥ 65 years	3	3.2	39,794	89.1	0.01
Total	91	100.0	44,628	100.0	0.20

Table 2 presents the HIV/AIDS-specific mortality rates stratified by sex, age group, study period, and DL. For this analysis, DL was categorized into two groups: low deprivation level (DI ≤ P75, encompassing DL1, DL2, and DL3) and high deprivation level (DI > P75, encompassing DL4 and DL5). Overall, men consistently had higher HIV/AIDS mortality rates than women across most age groups, DLs, and study periods. Mortality rates were generally higher in CTs with high deprivation, but the trends between periods were heterogeneous. For example, among men aged 0-44 in high deprivation areas, mortality decreased substantially from the first to the second period (from 20.76 [95%CI: 17.44-24.08] to 4.97 [95%CI: 3.32-6.62]), while in men aged 45-64 with high deprivation, mortality increased (from 14.47 [95%CI: 9.74-19.20] to 18.60 [95%CI: 13.49-23.70]). Among women, mortality declined in the 0-44 age group (from 6.33 [95%CI: 4.42-8.25] to 4.08 [95%CI: 2.51-5.64]) but increased in the 45-64 age group, particularly in areas with high deprivation (from 5.24 [95%CI: 2.50-7.99] to 6.67 [95%CI: 3.67-9.67]). These contrasting patterns highlight that changes over time were not uniform and depended on age, sex, and DL.

**Table 2:** Specific mortality rates (x 100,000) due to HIV/AIDS, according sex, age group, deprivation level (DL), and period. Cities of Alicante, Castellón and Valencia, Spain, 2000-2015.

Sex/Age group/DL *	2000-2007		2008-2015	
	Rate	95%CI	Rate	95%CI
Male				
0-44 years				
Low	7.25	6.15-8.35	2.45	1.81-3.09
High	20.76	17.44-24.08	4.97	3.32-6.62
45-64 years				
Low	9.76	7.66-11.86	8.69	6.88-10.51
High	14.47	9.74-19.20	18.6	13.49-23.70
≥ 65 years				
Low	2.89	1.37-4.40	1.76	0.67-2.86
High	5.23	1.81-8.65	4.59	1.41-7.77
Female				
0-44 years				
Low	3.19	2.45-3.92	0.79	0.43-1.16
High	6.33	4.42-8.25	4.08	2.51-5.64
45-64 years				
Low	1.15	0.47-1.83	2.21	1.34-3.07
High	5.24	2.50-7.99	6.67	3.67-9.67
≥ 65 years				
Low	0.13	0.00-0.40	0.12	0.00-0.35
High	0.40	0.00-1.19	0.77	0.00-1.84

95%CI: 95% confidence interval.

* Deprivation level of the census tract of residence based on the deprivation index (DI). Low deprivation level: DI ≤ P75; high deprivation level: DI > P75.

Figure 1 illustrates the specific mortality rates by sex, age group, and DL. In the 0-44 age group, both men and women, and across all DLs, experienced a clear decrease in HIV/AIDS mortality rates from the first to the second period. In contrast, in the 45-64 and ≥ 65 groups, changes between periods were less consistent. Across all age groups, a common pattern was observed in which HIV/AIDS mortality rates increased with higher deprivation, with the highest levels systematically found in DL5.

Table 3 shows the RR of death by DL, stratified by age, sex, and period. Across all age groups and periods, higher DLs were consistently associated with an increased risk of HIV/AIDS death for both men and women. The RR were significantly greater (p < 0.05) than 1 in most groups analyzed, particularly among men aged 0-44 years (2000-2007: RR = 2.863; 95%CI: 2.296-3.569; 2008-2015: RR = 2.028; 95%CI: 1.332-3.090) and women aged ≥ 45 years (2000-2007: RR = 4.137; 95%CI: 1.937-8.838). Furthermore, the RR between DL categories did not show a general decline from the first to the second period.

**Table 3:** Relative risks (RR) of for death due to HIV/AIDS deaths according to deprivation level (DL), specific for age, sex, and period.

Sex/Age group/DL *	2000-2007		2008-2015	
	RR	95%CI	RR	95%CI
Male				
0-44 years				
High	2.863	2.296-3.569	2.028	1.332-3.090
Low	1.000		1.000	
≥ 45 years				
High	1.472	1.034-2.097	2.120	1.535-2.928
Low	1.000		1.000	
Female				
0-44 years				
High	1.987	1.358-2.907	5.137	2.817-9.370
Low	1.000		1.000	
≥ 45 years				
High	4.137	1.937-8.838	2.932	1.650-5.211
Low	1.000		1.000	

95%CI: 95% confidence interval.

* Deprivation level of the census tract of residence based on the deprivation index (DI). Low deprivation level: DI ≤ P75; high deprivation level: DI > P75.

Table 4 shows the RR of death for the 2008-2015 period compared to 2000-2007, stratified by sex, age, and DL. In men, there was a clear reduction in HIV/AIDS mortality risk in the 0-44 age group across both DLs (low deprivation: RR = 0.338; 95%CI: 0.250-0.466; high deprivation: RR = 0.239; 95%CI: 0.166-0.346), while no significant changes were observed among those aged ≥ 45 years (low deprivation: RR = 0.845; 95%CI: 0.645-1.131; high deprivation: RR = 1.230; 95%CI: 0.835-1.813). Among women, the strongest reduction was observed in the 0-44 age group with low deprivation (RR = 0.249; 95%CI: 0.149-0.417), while those aged ≥ 45 years showed a tendency towards increased risk in both low and high deprivation categories (low deprivation: RR = 1.874; 95%CI: 0.945-3.713; high deprivation: RR = 1.328; 95%CI: 0.685-2.576). Overall, the largest reductions in HIV/AIDS mortality risk were observed among younger individuals, while in older groups the changes were heterogeneous and generally not statistically significant (p > 0.05).

**Table 4:** Relative risks (RR) for HIV/AIDS deaths in 2008-2015 vs. 2000-2007, specific to age, sex, and deprivation level (DL).

DL */Age group	Men		Women	
	RR	95%CI	RR	95%CI
Low				
0-44 years	0.338	0.250-0.456	0.249	0.149-0.417
≥ 45 years	0.854	0.645-1.131	1.874	0.945-3.713
High				
0-44 years	0.239	0.166-0.346	0.249	0.039-1.050
≥ 45 years	1.230	0.835-1.813	1.874	0.685-2.576

95%CI: 95% confidence interval.

* Deprivation level of the census tract of residence based on the deprivation index (DI). Low deprivation level: DI ≤ P75; high deprivation level: DI > P75.

## Discussion

This study demonstrated that HIV/AIDS mortality inequalities between areas of high and low deprivation in the large cities of the Valencian Community persisted even after the onset of the economic crisis. The proportion of deaths due to HIV/AIDS remained higher in areas with greater deprivation, and the analysis of mortality rates and relative risks confirmed a consistently higher mortality risk in these areas. Furthermore, while overall HIV/AIDS mortality decreased from the first to the second period, this trend showed exceptions depending on DL, age group, sex, and period.

During the 2008 economic crisis, two opposing trends in HIV/AIDS mortality were observed. The first, known as the countercyclical trend, arises from increased poverty, leading to higher levels of morbidity and mortality in the population ^23^. Conversely, the procyclical trend occurs during macroeconomic downturns when the promotion of certain healthy behaviors improves living conditions for some segments of the population ^24^. However, despite a potential overall procyclical trend following a recession, mortality may still rise in specific social sectors or geographic areas. Additionally, it is noteworthy that mortality inequalities persisted after the economic crisis, with no general increases observed except among certain groups, stratified by sex and age ^22^.

HIV/AIDS mortality declined markedly among younger adults (aged 0-44 years) across all DLs. In contrast, among individuals aged ≥ 45 years, mortality remained stable or showed a slight increase, particularly among women with low deprivation and among both sexes in highly deprived areas, although these differences were not statistically significant. These trends may be explained by a lower incidence of new infections and improvements in antiretroviral treatments, which have shifted the risk of HIV/AIDS mortality to older age groups. For instance, a meta-analysis by Poorolajal et al. ^25^ found that survival after the onset of AIDS for patients receiving HAART exceeds 10 years, compared to just two years for those not receiving HAART. Therefore, a potential hypothesis is that the age of mortality may be gradually shifting to older individuals, as the improved survival rates resulting from treatment extend the lifespan of people living with HIV/AIDS. This suggests that advances in treatment may be contributing to the observed rise in mortality in older age groups. Moreover, the increase observed among adults aged 45 years and older in areas of high deprivation may reflect cohort effects of individuals infected in earlier decades, who reached middle age with accumulated comorbidities and faced greater barriers to sustained access and adherence to antiretroviral therapy. This interpretation is consistent with the persistence of social inequalities in HIV/AIDS mortality.

Numerous studies have established a relationship between socioeconomic status and HIV/AIDS mortality, indicating a higher risk of death in areas with lower socioeconomic status ^9^. Furthermore, some of these studies suggest that individuals with HIV who experience higher levels of deprivation are less likely to have access to crucial information and treatment ^9^, both of which are critical factors in managing the progression of the disease. Another potential explanation for the observed association between HIV/AIDS mortality and socioeconomic status is the link between the disease and unhealthy lifestyle behaviors, which are more prevalent in areas of greater deprivation. These behaviors, including substance use, particularly the injection of drugs, and high-risk sexual practices, are known to exacerbate the spread and progression of HIV ^26^ ^,^ ^27^.

The findings of this study show that, in both men and women across both periods, there is an elevated risk of mortality in CTs with a high level of deprivation. Notably, HIV/AIDS mortality is higher in men than in women, with global data from 2020 reporting that 58% of all HIV infections occurred in men. Furthermore, men are less likely than women to be aware of their HIV status, to receive treatment, and to achieve viral suppression ^1^. In the three cities of the Valencian Community, men consistently exhibit higher HIV/AIDS-related mortality rates than women across all age groups, DL categories, and time periods. Gender norms, such as the denial of vulnerability often associated with heterosexual men, may contribute to these disparities ^28^. Additionally, structural factors, such as barriers to accessing sexual health care services for men, should be considered as potential contributors to these outcomes ^29^.

In both study periods, the highest HIV/AIDS mortality risks were observed in women residing in high-deprivation areas, suggesting significant gender interactions with mortality outcomes. Previous studies have highlighted that factors such as low educational attainment and ethnicity may interact with gender to increase women’s vulnerability to the disease ^30^ ^,^ ^31^. Thus, different social groups may follow gender norms and behaviors that negatively impact women’s health. Puskas & Hogg ^32^ emphasize the importance of considering gender as a key variable in public health interventions, as gender differences influence treatment adherence and outcomes. They argue that interventions should be tailored to specific subgroups, considering sociocultural factors and social support systems.

This study has some limitations, including those inherent to an ecological study design. As with all ecological studies, the ecological fallacy must be considered, as associations observed at the aggregated level cannot be assumed to hold for individuals. Furthermore, the use of CTs as the unit of analysis introduces additional constraints related to spatial aggregation, such as the modifiable areal unit problem (MAUP), and limits the ability to capture within-area heterogeneity in socioeconomic conditions. In addition, as CTs are used as the unit of analysis, the relationship between DL and HIV/AIDS mortality risk at the individual level cannot be assessed. A further limitation is the lack of georeferencing for some deaths; however, only 27 deaths (2.8%) could not be georeferenced, a percentage that is much lower than those typically found in similar studies, suggesting minimal impact on the results. Furthermore, the number of HIV/AIDS deaths among older adults was relatively low, resulting in wide confidence intervals and requiring cautious interpretation. Therefore, age group categories were regrouped into two broader intervals (0-44 and ≥ 45 years) to improve statistical stability while maintaining comparability with previous studies. Finally, although alternative model specifications such as negative binomial or zero-inflated models could also be considered, exploratory analyses indicated that the results were consistent across specifications. Therefore, and to preserve parsimony and stability in the estimates given the limited number of deaths when stratified, we present Poisson regression with robust estimation as the main approach.

## Conclusion

This study confirms that HIV/AIDS mortality inequalities persist across both periods, despite an overall decline in mortality. Mortality trends since the onset of the crisis generally followed a procyclical pattern; however, certain population groups show a countercyclical trend, including men aged ≥ 45 years with high DLs and women in the same age group.

To improve population health and progress toward the eradication of HIV/AIDS, it is crucial to address the barriers to prevention and treatment faced by vulnerable groups. A primary policy objective should be the reduction or elimination of health care inequalities. Public health care strategies should be grounded in the social determinants of health, prioritizing targeted interventions for key populations at risk, such as those in high-deprived areas. Future research should further investigate the social determinants of health in these high-deprivation areas to better understand the contextual factors and behaviors that contribute to higher HIV/AIDS mortality.

## Data Availability

The databases used in the study, including extraction codes, analyses, and results, are available in the repositories: https://www.ine.es/ (Spanish National Institute of Statistics; all population, and mortality data) and https://www.uv.es/medea/medeapp.html (MEDEA-3 project database; georeferenced data).
