# Supplementary material for: Socioeconomic inequalities in HIV/AIDS mortality in urban areas of three Spanish cities
Source: Cad Saude Publica. 2026 Mar 23;42:e00141925. doi: 10.1590/0102-311XEN141925 (PMC13011258; doi:10.1590/0102-311XEN141925)
Supplement: Supplementary Material [file 1678-4464-csp-42-EN141925-s.pdf]

SUPPLEMENTARY MATERIAL

Table S1 Average population per year in the cities under study according to period, age group, sex, and deprivation level.

Period	(DL)[a]	0 -44		45 -64		≥ 65	
		Men	Women	Men	Women	Men	Women
**2000-2007**	DL1	27649	28172	11642	14050	6802	11100
	DL2	61455	61018	23269	26272	12625	19926
	DL3	198787	193125	71408	79503	41207	62468
	DL4	55670	51990	20026	21407	12805	18065
	DL5	34655	30895	11070	11989	8705	12994
	Total	378213	365200	137416	153219	82144	124552
**2008-2015**	DL1	23122	23128	11377	13725	7600	12044
	DL2	69293	68923	29524	33207	15390	22695
	DL3	198369	191465	85647	94619	47885	70375
	DL4	52128	48464	21396	22664	13292	19839
	DL5	35897	31253	12886	12951	8486	12493
	Total	378808	363229	160829	177164	92650	137443

(a) DL: Deprivation level in the census tracts of residence, based on the DI. DL1: $DI<P_{10}$; DL2: $P_{10} \leq DI < P_{25}$; DL3: $P_{25} \leq DI < P_{75}$; DL4: $P_{75} \leq DI < P_{90}$; DL5: $DI \geq P_{90}$; P_q= Percentil q

Table S2 Relative risks (RR) of death due to HIV/AIDS according to deprivation level (and 95% confidence intervals, 95%CI), specific for age, sex, and period.

Sex	Age	Deprivation level[a]	2000-2007			2008-2015		
			RR	95% CI		RR	95% CI	
				Lower	Upper		Lower	Upper
Men	0-44	High	2.863	2.295	3.568	2.028	1.320	3.073
		Low	1	.	.	1	.	.
	45-64	High	1.483	0.992	2.174	2.139	1.506	3.007
		Low	1	.	.	1	.	.
	≥65	High	1.812	0.755	4.131	2.604	0.994	6.600
		Low	1	.	.	1	.	.
Women	0-44	High	1.987	1.348	2.892	5.137	2.835	9.517
		Low	1	.	.	1	.	.
	45-64	High	4.567	2.078	10.294	3.021	1.643	5.465
		Low	1	.	.	1	.	.
	≥65	High	3.010	0.119	76.052	6.502	0.623	139.816
		Low	1	.	.	1	.	.

(a) Deprivation level of the census tract of residence based on the deprivation index (DI). Low deprivation level: DI ≤ P_{75}, High deprivation level: DI > P_{75}

Table S3 Relative risks of death from HIV/AIDS in 2008-15 vs. 2000-07 (and 95% confidence intervals, 95%CI), specific to age, sex, and deprivation level.

Deprivation level[a]	Age	Men			Women		
		RR	95% CI		RR	95% CI	
			Lower	Upper		Lower	Upper
Low	0-44	0.338	0.248	0.453	0.249	0.144	0.408
	45-64	0.891	0.660	1.203	1.924	0.971	4.073
	≥65	0.611	0.263	1.366	0.889	0.035	22.471
High	0-44	0.239	0.163	0.341	0.644	0.390	1.042
	45-64	1.285	0.842	1.982	1.273	0.641	2.587
	≥65	0.878	0.330	2.296	1.921	0.184	41.312

(a) Deprivation level of the census tract of residence based on the deprivation index (DI). Low deprivation level: DI $\leq P_{75}$, High deprivation level: DI $> P_{75}$
